# Elbow terrible triad: Advancing current concepts in the treatment of 13 cases

**DOI:** 10.1016/j.tcr.2025.101146

**Published:** 2025-02-24

**Authors:** Edgar Barros Prieto, Eduardo Noboa, Carlos Ballesteros, Carlos Peñaherrera, Francisco Endara, Alejandro Barros Castro

**Affiliations:** a Department of Orthopedics and Traumatology, Hospital Voz Andes, Quito, Ecuador; b Department of Orthopedics and Traumatology, Hospital Metropolitano, Quito, Ecuador; c International University of Ecuador in the Metropolitan Hospital, Quito, Ecuador

**Keywords:** Elbow, Trauma, Fracture and dislocation, Terrible-triad injury, Treatment protocol, Coronoid process, Elbow approaches

## Abstract

**Background:**

The “terrible triad” of the elbow is characterized by a combination of elbow dislocation with fractures of the radial head and the coronoid process of the ulna. This injury is notoriously complex due to the rupture of key elements that ensure elbow stability, resulting in poorer outcomes compared to simpler joint injuries. Despite advances in treatment protocols, an optimal surgical approach and fixation technique standard have not yet been established.

**Methods:**

This descriptive study focused on reviewing the anatomical and clinical characteristics of the elbow joint, considering the most current biomechanical concepts. Management protocols implemented in our practice were evaluated, including specific surgical techniques and fixation methods based on a deep understanding of elbow anatomy and biomechanics. Retrospective data were collected from patients treated at our centers, analyzing postoperative clinical and radiographic outcomes.

**Results:**

The implementation of a treatment based on updated concepts of elbow biomechanics and anatomy showed a significant improvement in postoperative joint stability and functional recovery of patients. There was a reduction in complication rates and an improvement in clinical outcomes compared to traditional approaches. The selection of specific fixation techniques and surgical approaches allowed for more effective management of the “terrible triad,” adapting to the particularities of each case and optimizing results.

**Conclusion:**

This study highlights the importance of a detailed understanding of elbow biomechanics and anatomy for the effective management of the “terrible triad.” The implementation of protocols based on these principles can significantly improve clinical outcomes and reduce complications, offering a comprehensive approach to addressing this complex pathology.

Level of Evidence: IV

## Introduction

The “terrible triad” described by Hotchkiss [1] was known as the combination of elbow dislocation with fractures of the radial head and the coronoid process of the ulna. This injury was notable for its complexity, due to the rupture of elements that formed part of the various components contributing to elbow stability [2]. This was reflected in the commonly reported poor outcomes, especially in comparison with simpler elbow joint injuries [3]. Improved understanding of the injury mechanism, relevant anatomy, and factors influencing joint stability allowed the development of standardized treatment protocols [[4], [5], [6]]. Although these studies validated the efficacy of certain approaches [3], a unified reference standard had not yet been established to simplify the choice of the optimal surgical approach and fixation technique for this pathology. Therefore, the objective of this research was to delve into the unique anatomical characteristics of the elbow joint, considering the most current concepts to understand its biomechanics, and to report on the outcomes of applying these current concepts in their management. This approach aimed to comprehensively address this pathology and facilitate the selection of the appropriate surgical approach and the ideal fixation technique.

## Current concepts in the terrible triad of the elbow

### Structure and function

The function of the elbow was to allow the optimal positioning of the hand in space. The elbow was presented as a synovial hinge joint, comprising the humeroradial, radiocapitellar (RC), and ulnohumeral (UH) joints. These joints allowed the movements of flexion-extension and pronation-supination. It was widely accepted in the scientific literature that the normal range of motion for the elbow joint spanned from 0° to 140°, while a more restricted range, specifically from 30° to 130°, was required for performing daily activities [7].

### Osteology

The elbow joint was formed by three bones: the distal humerus, and the proximal radius and ulna. The distal humerus region displayed two distinct articulations: the trochlea and the capitulum. The trochlea, articulated with the trochlear notch of the ulna, had an anterior inclination of 30°. The proximal ulna featured the trochlear and radial notches, which exhibited highly congruent anatomy, allowing extensive articular contact during elbow movement. Both the olecranon and the coronoid process along with the trochlea provided stability within a 170° range, contributing significantly to bone stability, with soft tissues playing a secondary role [8]. The radial notch of the ulna articulated with the margin of the radial head at the proximal radioulnar joint. The radial head, an elliptically concave structure covered by articular cartilage over 270° enabling pronosupination [9], articulated with the capitulum and radial notch of the ulna. Additionally, it acted as an anterior buttress and static stabilizer against valgus stress when the elbow was flexed between 20° and 120°, providing up to 2° of rotational stability at the radio capitellar joint9^−10^. However, due to significant incongruence with the capitulum throughout its range of motion and covering only a 90° arc, soft tissues played a more critical role in the lateral region. The coronoid process-maintained elbow stability by sliding into the coronoid fossa of the humerus during flexion. This triangular protrusion was in the anterior proximal region and consisted of a tip, body, and two facets (anterolateral and anteromedial, separated by a bony bridge). The anteromedial facet, with a surface area of 232 mm^2^, acted as a primary stabilizer. The anterolateral facet played a role as a secondary valgus stabilizer, with a smaller surface area of less than 142 mm2, supported in this function by the radial head [9,10]. Elbow bone stability reached its maximum effectiveness during flexion due to the symmetrical positioning of the coronoid process and radial head within their respective articular fossae [[9], [10], [11]].

### Static and dynamic stabilizers

The anterior capsule played a role in providing valgus stability and joint proprioception [[9], [10], [11]]. The insertions of the anterior joint capsule proximally on the humerus were located in the radial fossa and coronoid fossa of the humerus, extending to the anterior aspect of the medial and lateral epicondyles. Distally, it attached to the coronoid process of the ulna and the annular ligament [11]. The insertion of the posterior joint capsule extended from the superior margin of the olecranon fossa to the non-process and the annular ligament [11]. The collateral ligaments of the elbow referred to thickenings of the joint capsule in the medial and lateral regions, contributing to increased stability in the joint [12]. The medial collateral ligament complex (CLM) of the elbow consisted of the medial ulnar collateral ligament (MUCL), which in turn comprised anterior, posterior, and oblique bundles also known as the ligament of Copper1 [3]. The MUCL acted as the primary static stabilizer within the range of flexion from 70° to 120°. The CLM resisted valgus stress forces and internal rotation primarily through tension on the anterior bundle of the MUCL; during flexion-extension, the anterior bundle was taut from 0° to 60°, with the posterior bundle tensioned from 60° to 120°. The humeral origin of the MUCL was 8.5 mm distal and 7.8 mm anterior to the medial epicondyle of the humerus [14], positioned 15 mm from the articular line with an area of 17 to 45 mm2. The ulnar insertion of the MUCL averaged 5.3 mm from the center of the sublime tubercle along the ulnar crest (5.8–7.8 mm from the articular line) [14]. The average length and width of the MUCL were 31.9 mm (range, 21.1–53.9 mm) and 5.95 mm (range, 4.5–7.6 mm), respectively [15,16].

The lateral ligament complex (LLC) consisted of three main ligaments: the lateral ulnar collateral ligament (LUCL), the radial collateral ligament (RCL), and the annular ligament (AL). The LLC served as the primary static stabilizer resisting external rotation forces [12]. During flexion-extension, while the RCL remained relatively isometric throughout the range of motion, the LUCL was laxer during elbow extension and tightened with flexion [12]. The LUCL acted as the principal posterolateral stabilizer of the elbow, also known as the ligament of Osborn Cotterill [17,18]. The humeral origin of the LUCL (more distal) and the RCL (more proximal) originated isometrically 8.2 mm from the distal articular surface and 7.3 mm from the anterior articular surface of the trochlea, separated by 1.1 mm on the inferior surface of the lateral epicondyle, with average areas of 26 mm^2^ and 31.6 mm^2^, respectively. The ulnar insertion of the LUCL was 3.3 mm from the apex of the supinator crest with an average area of 22 mm^2^ in the lateral cubital region, distal to 2 mm from the insertion of the AL. The distal insertion of the RCL was the largest of all ligaments due to its fan-like arrangement on the lateral surface surrounding the radial head, averaging 292 mm^2^. The AL encircled the radial head and attached to the anterior (insertion; average area of 36 mm^2^, 14 mm from the tip of the coronoid process) and posterior margins (origin; average area of 25 mm^2^) of the radial notch of the ulna [18,19].

### Definition

The “terrible triad of the elbow,” first described by Hotchkiss in 1996 [1], was characterized by its poor prognosis involving stiffness and instability, defined as a combination of radial head fracture, coronoid process fracture, and elbow joint dislocation [18], often associated with collateral ligament injuries. It commonly resulted from high- or low-energy trauma, with approximately 60 % occurring in accidents involving significant force, such as falls from height or motor vehicle accidents [19]. This injury was present in 80 % of posterior-lateral elbow dislocations. The injury was more likely under conditions of shoulder abduction, elbow extension, forearm supination, and axial load applied to the upper limb [20]. The injury-fracture pattern in the terrible triad was described by O'Driscoll et al. in 1992 as the “circle of Horri,” indicating a disruption process starting laterally (LLC), where force vectors divided anteriorly and posteriorly, leading to capsule failure and progressing medially (CLM) [21]. The stages were as follows: Stage 1: LLC disruption; Stage 2: Anterior and posterior capsule disruption; Stage 3: CLM disruption + Impaction fracture of the radial head and shear fracture of the coronoid process [22].

### Biomechanics

In understanding the “terrible triad,” it is crucial to analyze the structural role of each component in the injury. The humerus acted as a three-column structure: medial (including the anteromedial facet of the coronoid and medial trochlea), central (composed of the anterolateral facet of the coronoid and lateral trochlea), and lateral (referring to the humeral head). The pivot point for varus and valgus forces, located between the medial and central columns, specifically the coronoid process, constituted approximately 60 % of humeral support [15]. The coronoid process functioned through its two facets, with emphasis on the anteromedial facet due to its larger surface area. The anterior capsule inserted approximately 5 mm distal to the articular line, and the brachialis muscle reinforced the coronoid by inserting just distal to this point, providing some protection against fractures [[11], [12], [13], [14], [15], [16], [17]]. Watts concept allows understanding that the main restriction for valgus collapse is the lateral column, with a secondary contribution from the central column, while the main restriction for varus collapse is the medial column. The central column is not critical when the lateral column is functional and protected from adjacent column injuries. However, removing the lateral column makes the central column essential for valgus stability.

Therefore, for the “terrible triad” to occur, the previously mentioned mechanism must occur external rotation along with valgus. This process initially involves avulsion of the lateral collateral ligament complex, followed by fracture of the anterolateral region of the radial head (lateral column) and, finally, fracture of the anterolateral facet of the coronoid, associated with dislocation. According to Wrightington's classification of elbow dislocation fractures, the terrible triad corresponds to type C [17], characterized by disruption of the lateral collateral ligament complex resulting in valgus instability [17,18]. Thus, restoring the radial head (lateral column) achieves stability without necessarily fixing the anterolateral facet fracture of the coronoid (central column), provided the lateral ligament injury is repaired [[18], [19], [20], [21]]. Isolated fractures of the radial head are generally stable since only the lateral column is involved, but when there is fragmentation of the radial head, associated injury to lateral and medial soft tissue stabilizers may be sufficient to produce instability and may require lateral column reconstruction and soft tissue stabilization [[19], [20], [21], [22]].

## Materials and methods

A series of thirteen consecutive cases of elbow dislocation with radial head and coronoid process fractures were identified as the terrible triad at the Metropolitan Hospital of Quito and Voz Andes Hospital of Quito from July 2018 to December 2023. The inclusion criteria for the study were meticulously selected to ensure the homogeneity and quality of the obtained data. Patients with a confirmed diagnosis of terrible triad using computed tomography were included and followed longitudinally for a minimum of 2 years to evaluate their performance using the Mayo Elbow Performance Score (MEPS) and to assess elbow range of motion. Additionally, radiographic evaluations were conducted using the Broberg and Morrey classification to analyze bone consolidation and detect complications such as heterotopic ossification. Exclusion criteria encompassed patients with inadequate follow-up for comprehensive clinical evaluation, as well as cases treated with non-standardized methods potentially introducing uncontrolled variables into the study outcomes.

During this period, fracture consolidation and recovery of joint mobility ranges were observed. One patient was lost to follow-up prior to data analysis, leaving us with 12 patients (12 elbows) for evaluation. The sample included a total of 4 men and 9 women (69.2 %). Regarding the laterality of the injuries, 7 patients (53.8 %) had left elbow fractures. The average age was 62.8 years (range 26 to 80 years). Two preoperative complications (serous blisters) were presented, although these showed no correlation with the surgical resolution employed. Of the patients, 5 had comorbidities such as hypertension, consistent with the average age. The average surgery time was 3.2 h, while the average hospital stay was 2.4 days. Three patients did not have ligament injuries, while the rest required surgical resolution of these injuries. In 9 patients, the Wrightington approach [21] was used, when necessary, combined with other approaches.

**Fig. 1:**
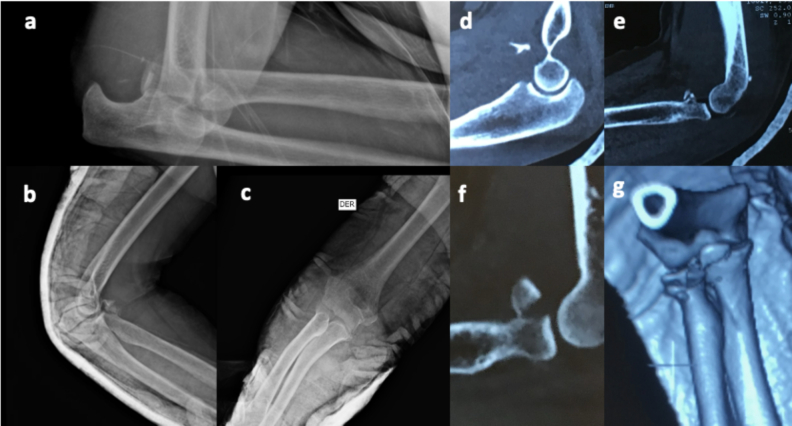
a) Lateral radiograph of the right elbow showing posterior elbow dislocation; b,c) lateral and AP radiographs respectively demonstrating reduction of dislocation + coronoid and radial head fractures; d,e,f) Sagittal cuts showing coronoid and radial head fractures; g) 3D reconstruction.

**Fig. 2:**
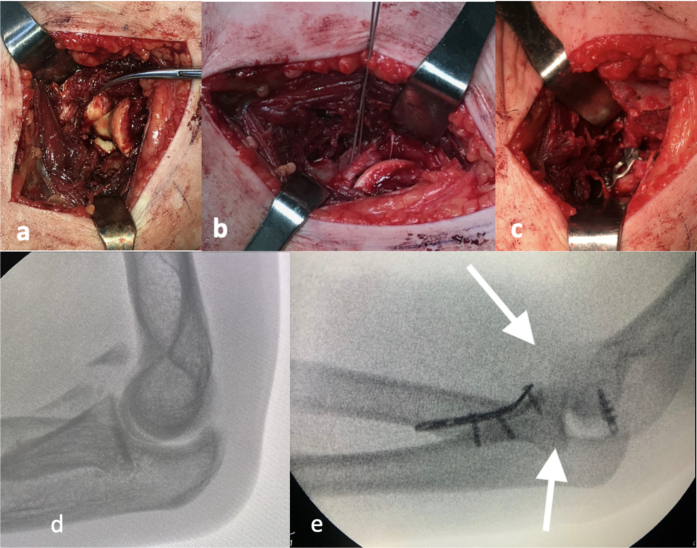
Anteromedial approach to address the coronoid process fracture. a) Exposure of the coronoid fracture; b) Preliminary reduction with K-wires; c) Placement of a metacarpal plate for reduction of the described fracture; d) Intraoperative fluoroscopy of the coronoid fracture; e) Intraoperative fluoroscopy of the coronoid fracture fixation.

Computed tomography was routinely used in all cases before surgery to identify patterns, comminution, and displacement not clear on radiographs (Fig. 1, Fig. 2). The O'Driscoll [23] classification was used for coronoid fractures, and the Mason [24] classification was used to classify radial head fractures. The average follow-up of the patients was 2 years, during which the clinical and radiological evolution of each patient was evaluated. The Mayo Elbow Performance Score (MEPS) [25] and elbow flexion-extension ranges were used for clinical evaluation at 2 months, 6 months, 1 year, and 2 years. The Broberg and Morrey [26] classification was used for radiographic evaluation to assess post-traumatic arthritis, and radiography allowed the evaluation of bone consolidation, and the identification of synostosis, heterotopic ossification, and joint congruence. Fractures, soft tissue injuries, and the type of material used can be seen in Table 1.

**Table 1:** Injury and treatment details.

Patient NO.	Radial head fracture		Coronoid fracture		Soft tissue injury		Approach
	Classification de Mason	ORIF	O'Driscoll	ORIF	Ligaments	Reparation	
1	II	Mini-fragment screw	II	MTC Hook Plate	LCL	Titanium Anchor	Wrightington+ anteromedial
2	III	MTC Hook Plate	I	–	LCL	Peek Anchor	Wrightington
3	III	Cupulectomy	I	–	LCL + MCL	Titanium Anchor	Wrightington
4	II	Mini-fragment screw	II	Mini-fragment Plate	LCL	Titanium Anchor	Wrightington+ anteromedial
5	II	Acutrak	II	Mini-fragment Plate	LCL+ MCL	Titanium Anchor	Anteromedial
6	I	–	III	MTC Hook Plate	–	–	Wrightinton + anteromedial
7	II	Mini-fragment screw	III	Mini-fragment screw	–	–	Wrightington+ anteromedial
8	I	–	III	MTC Hook Plate	LCM	High-Strength Suture	Wrightinton
9	I	–	III	Mini-fragment Plate	–	High-Strength Suture	Anteromedial
10	I	–	III	MTC Hook Plate	LCL+ LCM	High-Strength Suture	Wrightington+ anteromedial
11	II	Partial Cupulectomy	II	MTC Hook Plate	LCL+ LCM	Peek Anchor	Wrightington+ anteromedial
12	II	Mini-fragment plate	III	Mini-fragment screw	LCL	Peek Anchor	Wrightington+ anteromedial
13	III	MTC Hook Plate	I	No	LCL	Peek Anchor	Wrightington+ anteromedial

ORIF: Open Reduction and Internal Fixation; MTC: Metacarpal; LCL: Lateral Collateral Ligament; LCM: Medial Collateral Ligament.

### Surgical protocol

The 13 described cases were surgically intervened using primarily two approaches: The first was the posterolateral Wrightington approach [21], which allows addressing the radial head fracture. The second was the anteromedial approach to better expose coronoid fractures, especially the anteromedial facet (Fig. 2).

**Fig. 3:**
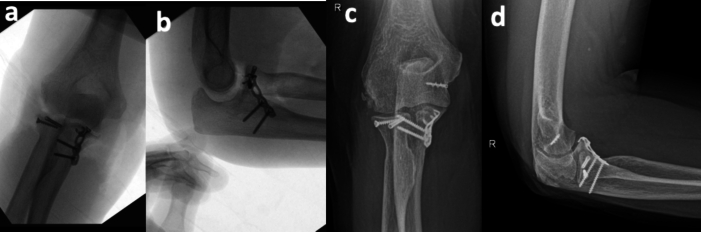
Patient case 1 Immediate intraoperative and postoperative images. a,b) AP and lateral fluoroscopic images showing adequate reduction but increased intra-articular space; c,d) AP and lateral radiographs with final result following repair of LCL and MCL.

The fixation order for all 13 patients was as follows: Fixation of the radial head, fixation of the ulnar fracture of coronoid process, and after evaluating intraoperative stability with dynamic tests, the decision was made whether to repair the soft tissues. Regarding the fixation of the radial head fracture, standard methods such as Acutrak screws and mini fragment screws were used, as well as unconventional methods like a 5th metacarpal hook plate or a specific distal radius fragment plate. These methods were chosen because non-conventional osteosynthesis has been shown in different studies to be very helpful for fixing specific fragments and achieving the necessary anatomical reduction for adequate consolidation, as demonstrated by Barros et al. [26] in their work on non-conventional osteosynthesis. In cases where anatomical reduction was not adequate, total or partial cupulectomy was opted [27]. The coronoid fracture fixation was performed using T-shaped anatomical plates as previously described non-conventional osteosynthesis. For soft tissue repair, anchors (Peek or Titanium) or high-strength sutures were used depending on the degree of injury identified during the intraoperative period (Fig. 3).

### Postoperative management

A cast was applied after surgery and used for 6 weeks in all patients. All patients had their elbows immobilized at 90° flexion with the forearm in pronation. This position aimed to prevent posterolateral instability and protect the lateral collateral ligament (LCL) repair. Supervised rehabilitation began on the second day post-surgery for all patients. Active flexion and extension exercise of the elbow and passive forearm rotation exercises with splint protection were performed for 20 min, 3 or 4 times a day, with a gradual increase in the range of motion (ROM). For example, the extension and flexion ROM of the elbow was set at 60°-110° during the first 2 weeks, then at 40°-120° during the next 4 weeks, and finally at 0°-130° degrees for 4 weeks, followed by the full range of elbow motion.

Celecoxib, 200 mg twice a day for 3 weeks postoperatively (21 days), was prescribed to prevent heterotopic ossification. All patients received acetaminophen 1000 mg three times a day, combined with tramadol drops, if necessary, to relieve pain and allow early active elbow exercise.

### Evaluation

The patients were evaluated clinically and radiographically for a maximum period of 2 years and a minimum of 6 months until the fracture united and the plateau stage of the elbow range of motion was reached. The function and stability of the elbow joint, as well as pain, were assessed, and the results were recorded. The Mayo Elbow Performance Score (MEPS) was determined for each patient at the final clinical visit. Radiography was used to assess bone consolidation, and to identify synostosis, heterotopic ossification, joint congruence, and the degree of post-traumatic arthritis. The obtained data can be seen in Table 2.

**Table 2:** Functional recovery and complications.

Patient no.	2 months		6 months		1 year		2 years		Broberg y Morrey classification	Synostosis	Heterotopic ossification
	ROM	MEPS	ROM	MEPS	ROM	MEPS	ROM	MEPS			
1	F:120° E:15°	80	F:160° E:0°	100	F:160° E:0°	100	F:160° E:0°	100	0	NO	NO
2	F:100° E:50°	65	F:100° E:20°	85	F:160° E:0°	100	F:160° E:0°	100	1	NO	NO
3	F: 90° E: 40°	65	F:100° E:20°	85	F:160° E:0°	100	F:160° E:0°	100	0	NO	NO
4	F:120° E:5°	70	F:100° E:20°	85	F:160° E:0°	100	F:160° E:0°	100	0	NO	NO
5	F:120° E:5°	70	F:100° E:20°	85	F:160° E:0°	85	F:160° E:0°	100	0	NO	NO
6	F: 90° E: 50°	65	F:120° E:20°	70	–	–	–	–	0	NO	NO
7	–	–	–	–	–	–	–	–	0	NO	NO
8	F: 90° E: 70°	55	F: 90° E: 50°	65	–	100	–	–	1	NO	NO
9	F:120° E:0°	100	F:160° E:0°	100	F:160° E:0°	100	–	–	0	NO	NO
10	F:120° E:0°	100	F:160° E:0°	100	F:160° E:0°	100	–	–	0	NO	NO
11	F:120° E:0°	100	F:160° E:0°	100	F:160° E:0°	–	–	–	0	NO	NO
12	F:100° E:50°	65	F:160° E:0°	80	–	–	–	–	0	NO	NO
13	F:100° E:50°	65	F:140° E:20°	80	–	–	–	–	0	NO	NO

ROM: Range of Motion; F: Flexion; E: Extension; MEPS: Mayo Elbow Performance Score.

## Results

**Fig. 4:**
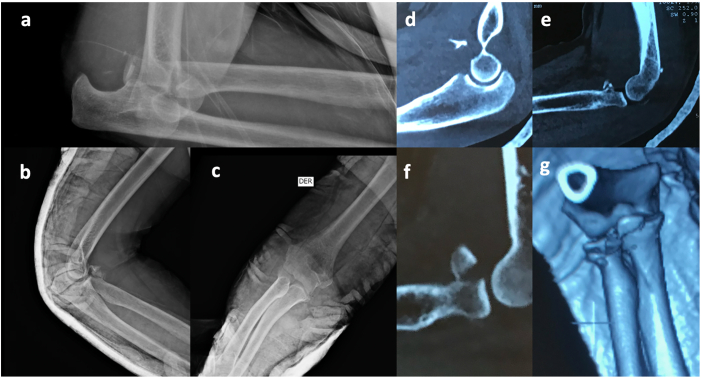
a) AP X-ray of the right elbow with “terible triad”; b-c) AP and Lateral X-ray after reduction; d-e-f-g) TC of “Terible Triad”.

A retrospective analysis of 13 cases of elbow fractures, sequentially collected, with a mean age of 62.8 years, was conducted. Of the total, 9 patients were women (69.2 %), suggesting a higher prevalence of this pathology in females. All female patients had suffered falls from standing height, correlating with existing literature describing this trauma mechanism as prevalent in this population. The average follow-up of the patients was 2 years. During this period, fracture consolidation and recovery of joint mobility ranges were observed. Two preoperative complications were recorded; however, these did not show a correlation with the applied surgical resolution. Five patients had comorbidities such as hypertension, consistent with the mean age (Fig. 3, Fig. 4).

Regarding the laterality of the injuries, 7 patients (53.8 %) had left elbow fractures, suggesting a tendency to protect the dominant side during trauma. The average surgery time was 3.2 h, while the average hospital stay was 2.4 days. Three patients did not have ligament injuries, while the rest required surgical intervention to resolve these injuries. In 7 patients, the Wrightington approach was used, when necessary, combined with other approaches. A correlation of 0.73 was found between the presence of comorbidities and increased hospital stay, suggesting that patients with underlying pathologies experienced longer hospitalizations due to the additional management required for their pre-existing conditions.

**Fig. 5:**
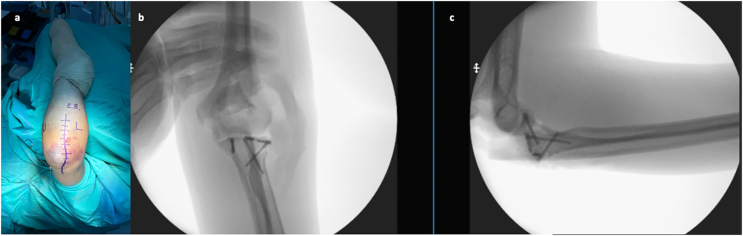
a) Wrightington posterolateral approach demarcation; b.c) AP and Lateral trans-surgical fixati.

The Wrightington approach showed a correlation of 0.82 with the recovery of mobility arcs, indicating that this approach facilitated a more effective surgical resolution and contributed to better functional recovery. Additionally, a correlation of 0.78 was observed between the Wrightington approach and surgical time, suggesting that this approach, by providing better visualization of the surgical field, allowed for a faster intervention, reducing surgery time and consequently, patient risk and hospital stay duration (Fig. 5).

In terms of mobility, at 2 months follow-up, the average flexion achieved was 107.5° and the average extension was 27.9°. The average Mayo scale score was 75. At 6 months, the average flexion increased to 130° and extension improved to 15°, with a Mayo score of 86.25. At one year, the average flexion achieved was 160° and extension 0°, with a Mayo score of 98.3. At two years of follow-up, the mobility ranges were maintained, with flexion of 160° and extension of 0°, and a Mayo score of 100, indicating an almost complete recovery of joint function.

The analysis revealed a multiple correlation of 0.82 between the Wrightington approach, hospital stay, surgical time, mobility arcs, and Mayo scale score. This suggests that the Wrightington approach, by reducing surgical time, also contributed to a shorter hospital stay, thereby enhancing the patients' ability to participate in physiotherapy and recover more effectively. The positive relationship between the surgical approach and functional outcomes highlights the importance of appropriate surgical approach selection to optimize patient recovery. In summary, the results indicate that the surgical technique employed was associated with efficient surgical resolution, shorter surgical time, and faster and more complete functional recovery in patients with elbow fractures. These findings underscore the procedure's efficacy in the surgical management of the terrible triad of the elbow, suggesting its preferential use in similar cases to improve clinical and functional outcomes.

## Discussion

The purpose of this study was to report the outcomes of implementing a treatment protocol used to address the terrible triad of the elbow. The results from the 13 cases demonstrated good outcomes with minimal surgical and postoperative morbidity. The major findings of the study were: (1) The combination of the Wrightington approach with the anteromedial approach instead of a posterior approach was effective for treating the terrible triad of elbow injuries. This method correlated with reduced surgical time, shorter hospital stays, and improved patient ability to participate in physiotherapy and recover more effectively. (2) The surgical procedures of this protocol significantly differed from traditional methods. The use of unconventional osteosynthesis techniques, by adapting plates originally designed for other anatomical regions, allowed for more precise anatomical reduction of fractured fragments in both the radial head and coronoid process. This innovation increased the available options for osteosynthesis in these specific anatomical areas. Additionally, this type of osteosynthesis did not compromise the outcome and considerably contributed to patient stability and functional recovery. (3) The sequence in addressing the pathologies constituting the terrible triad contributed to establishing joint complementarity, optimizing the surgical procedure. This approach not only standardized medical practice but also significantly reduced the duration of the intervention. As a result, patients experienced short and mid-term benefits, such as shorter hospital stays and earlier initiation of physiotherapy, thereby improving their postoperative recovery.

With the improvement of surgical strategies for the terrible triad of elbow injuries, excellent results have been reported in treatment [[28], [29], [30], [31]]. The outcomes achieved with our modified surgical method are equal to or better than those of other studies mentioned in the literature. Despite standardized treatment algorithms, patients with complex fracture-dislocation of the elbow frequently suffer from poor postoperative elbow function, leading to reduced quality of life. Jakobi et al. [32] identified factors associated with poor postoperative outcomes, determining that age over 70 years and BMI greater than 35 kg/m^2^ are significant risk factors for poor postoperative outcomes. Although good functional results are achieved in most cases with standardized treatment protocols, complication and revision rates are high, especially in these risk groups. The literature reports that the surgical protocol for the terrible triad of elbow injuries is well established as follows [[33], [34], [35]]: (1) Use a posterior approach for exposure; (2) Reduce and fix the coronoid fracture first; (3) Prefer the use of a metallic prosthesis over open reduction and internal fixation (ORIF) for radial head fracture; (4) Repair the lateral collateral ligament (LCL) complex and the common extensor origin and/or posterolateral capsule to restore lateral stability; and (5) Apply an articulated external fixator if residual joint instability persists. Although this treatment protocol has proven effective, instability, contracture, repair, and progression to osteoarthritis remain significant problems.

In our case series, we avoided using the posterior approach as it increases the risk of hematoma formation, heterotopic ossification, and flap necrosis [33]. Instead, we preferred the combination of the Wrightington and anteromedial approaches, which are less traumatic and more effective for exposure. The radial head fracture and LCL repair were performed via the Wrightington approach due to the mechanism of injury described in the literature, indicating that the capsuloligamentous structures of the elbow begin to fail from lateral to medial. Therefore, the reconstruction of the radial head and the LCL complex helps restore elbow joint stability, facilitating coronoid fracture fixation. The radial head is an important secondary stabilizer against valgus load and posterior translation. Although radial head replacement is typically performed, we prefer to repair the radial head rather than excise it or replace it with a prosthesis. The disadvantages of radial head plate fixation include the risk of posterior interosseous nerve injury, postoperative loss of forearm rotation, nonunion, and implant failure [34]. However, in our series, the complication rate was nil.

Despite its small size, the coronoid process is an important bony stabilizer of the humeroulnar joint. Posterior dislocation of the ulna relative to the distal humerus can be adequately reduced by stable fixation of the coronoid, achieving a congruent joint. Various surgical fixation techniques for the coronoid have been proposed. The approach used for coronoid fixation involves an anteromedial skin incision starting from the medial humeral epicondyle and following the axis of the flexor-pronator, then utilizing the “over-the-top” approach described by Hotchkiss [36]. A precise and stable internal fixation is easily achieved. Garrigues et al. [37] examined different surgical fixation techniques for coronoid fractures in cases of elbow fracture-dislocation, reporting that the suture loop technique was more stable than other techniques and had a lower complication rate. However, in our case series, we demonstrated the efficacy of unconventional fixation techniques, emulating the results of applying plates designed for other anatomical sites, yielding good results in terms of reduction and consolidation.

Once the restoration of the bony structure is completed, soft tissue injuries should be repaired. The LCL complex can be definitively reinserted into the lateral epicondyle with suture anchors or trans osseous sutures. The reason we perform LCL complex fixation as the last step is that the humeroulnar joint is generally dislocated or unstable before coronoid fracture reduction and fixation. After coronoid fracture fixation, the elbow is more stable and anatomically correct, at which point it is more appropriate to perform definitive LCL complex repair using the suture anchor technique. This method helps adjust the tension of the LCL complex to avoid laxity or over tension. The elbow stability is then evaluated using the hanging arm test and valgus stress test. If posterolateral instability persists from approximately 30° to full flexion in one or more forearm rotation positions or on intraoperative radiographs, the bone and soft tissue repairs are examined, and if evident valgus instability exists, the medial collateral ligament (MCL) is explored and repaired.

We initiate active rehabilitation exercises on the second postoperative day, routinely using an articulated plastic orthosis. With direct repair of the LCL complex, MCL, and bony structure, the elbow presents sufficient stability to allow immediate active-assisted movement. The muscle strength of the elbow flexors and extensors reinforces joint stability through dynamic stabilization. A biomechanical study demonstrated [38] that elbows with MCL and LCL injuries should be rehabilitated using active movements in a horizontal or vertical orientation.

There are certain limitations to this study that must be considered. Firstly, the sample size of patients was relatively small, which may affect the generalizability of the results. Additionally, all surgeries were performed by a single surgeon or under their direct supervision. This implies that the obtained results may not be reproducible by other surgeons or in different medical centers. However, it is important to highlight that the results indicated that the employed technique provides favorable outcomes with minimal morbidity.

## Conclusions

This study highlighted that, with a standardized surgical approach and innovative osteosynthesis techniques, excellent results could be achieved in the treatment of the terrible triad of the elbow. The combination of described approaches and techniques significantly improved functional recovery and reduced postoperative morbidity. The implementation of this protocol may establish a standard for the treatment of these complex injuries, offering an effective and enhanced solution for affected patients. Continued studies with larger samples and prolonged follow-up are recommended to further validate and refine these findings, ensuring that the proposed protocol remains the most effective in managing the terrible triad of the elbow.

## CRediT authorship contribution statement

**Edgar Barros Prieto:** Resources, Investigation, Conceptualization. **Eduardo Noboa:** Writing – review & editing. **Carlos Ballesteros:** Data curation. **Carlos Peñaherrera:** Writing – review & editing, Methodology, Conceptualization. **Francisco Endara:** Methodology, Data curation. **Alejandro Barros Castro:** Writing – review & editing, Writing – original draft, Methodology, Investigation, Conceptualization.

## Declaration of competing interest

We are pleased to submit our manuscript titled “ Elbow terrible triad: Advancing current concepts in the treatment of 13 cases” for consideration and potential publication in “ Trauma Case Reports”. This study provides a comprehensive analysis of our treatment protocols and outcomes in thirteen cases of the terrible triad of the elbow, highlighting current concepts and advances in management.

Our team adheres strictly to the highest ethical and legal standards in medical research. We confirm that all procedures conducted in this study were in accordance with the ethical guidelines established by our intitutions (Hospital Metropolitano Quito – Ecuador y Hospital Voz Andes Quito - Ecuador) and all patients provided informed consent prior to their participation.

We believe that the findings presented in this work are clinically relevant and will significantly contribute to the body of knowledge on the management of the terrible triad of the elbow. We appreciate the opportunity to be considered for publication in “ Trauma Case Reports” Journal.
